# Supplementary figures and images for: The gammaherpesviral TATA-box-binding protein directly interacts with the CTD of host RNA Pol II to direct late gene transcription
Source: PLoS Pathog. 2020 Sep 4;16(9):e1008843. doi: 10.1371/journal.ppat.1008843 (PMC7498053; doi:10.1371/journal.ppat.1008843)

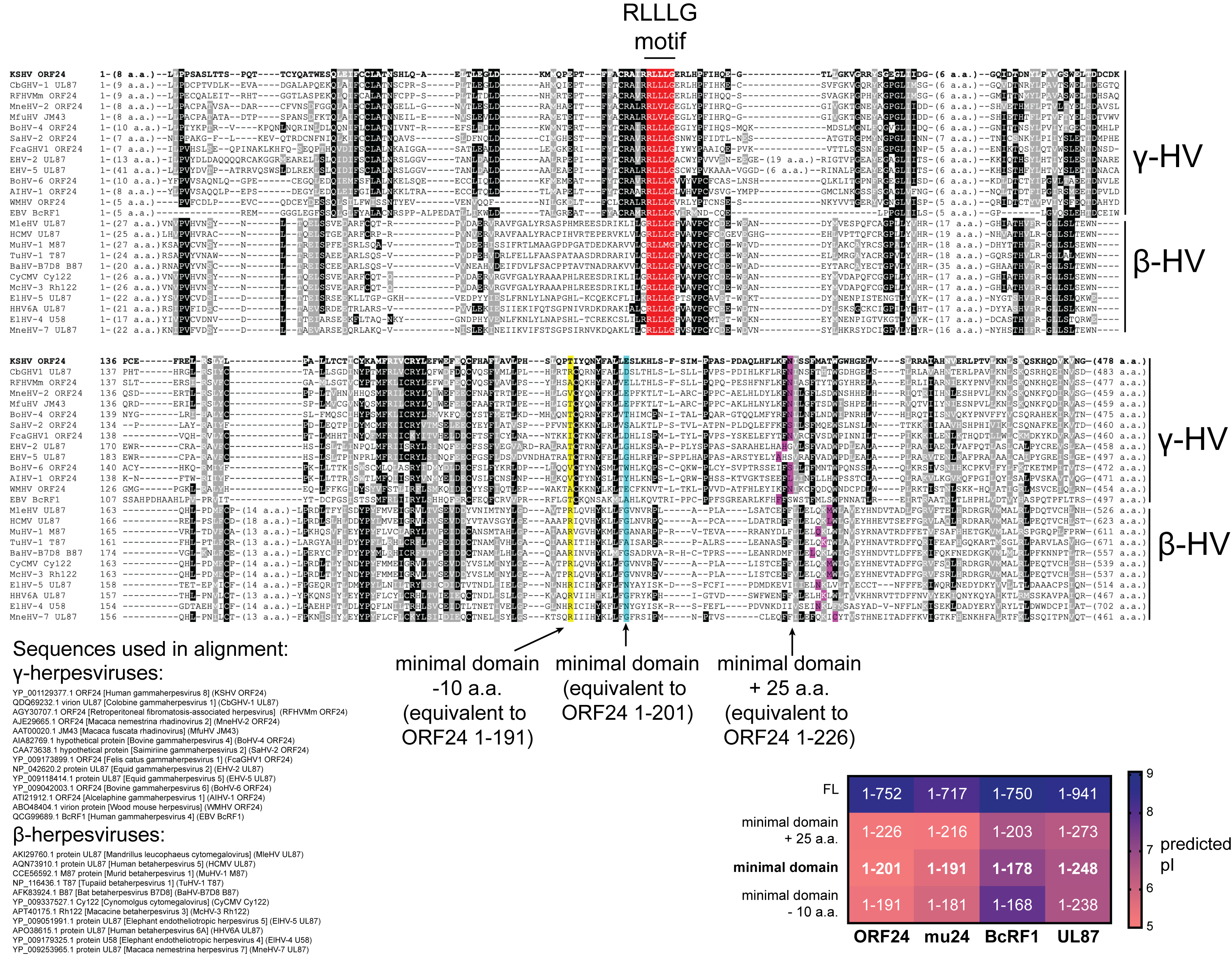

Supplement: S1 Fig — The conserved triple leucine motif is highlighted in red. The location of truncations for the constructs used in Fig 3 are highlighted in yellow, teal, and purple. Sequences used to construct the alignment are listed in the bottom left. The boundaries for the truncated constructs and their relative predicted isoelectric point based on [27] is shown in the bottom right. (TIF) [file ppat.1008843.s001.tif]

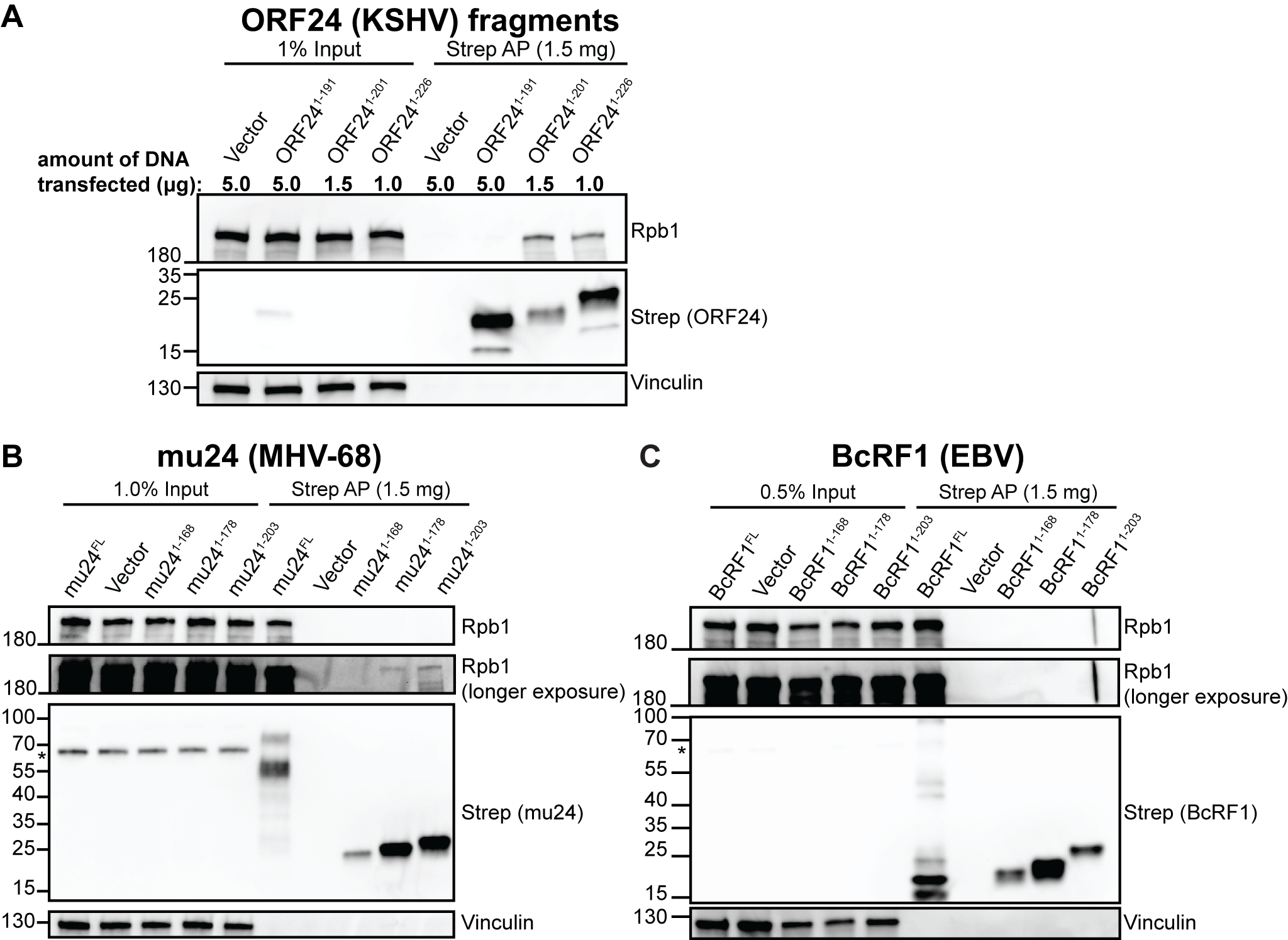

Supplement: S2 Fig — (*) indicates the presence of a non-specific band seen while using the anti-Strep antibody. (A) Truncated Strep-tagged constructs of ORF24 were used for the transient transfection/AP experiment. In all cases, 5 μg of total plasmid DNA was transfected. For the ORF24 a.a. 1–201 and 1–226 constructs, 1.5 and 1.0 μg of plasmid DNA was used with 3.5 and 4.0 μg of empty vector DNA, respectively. (B) Full-length or truncated Strep-tagged constructs of homologs from MHV68 (mu24) were used for the transient transfection/AP experiment. (C) Full-length or truncated Strep-tagged constructs of homologs from EBV (BcRF1) were used for the transient transfection/AP experiment. (TIF) [file ppat.1008843.s002.tif]

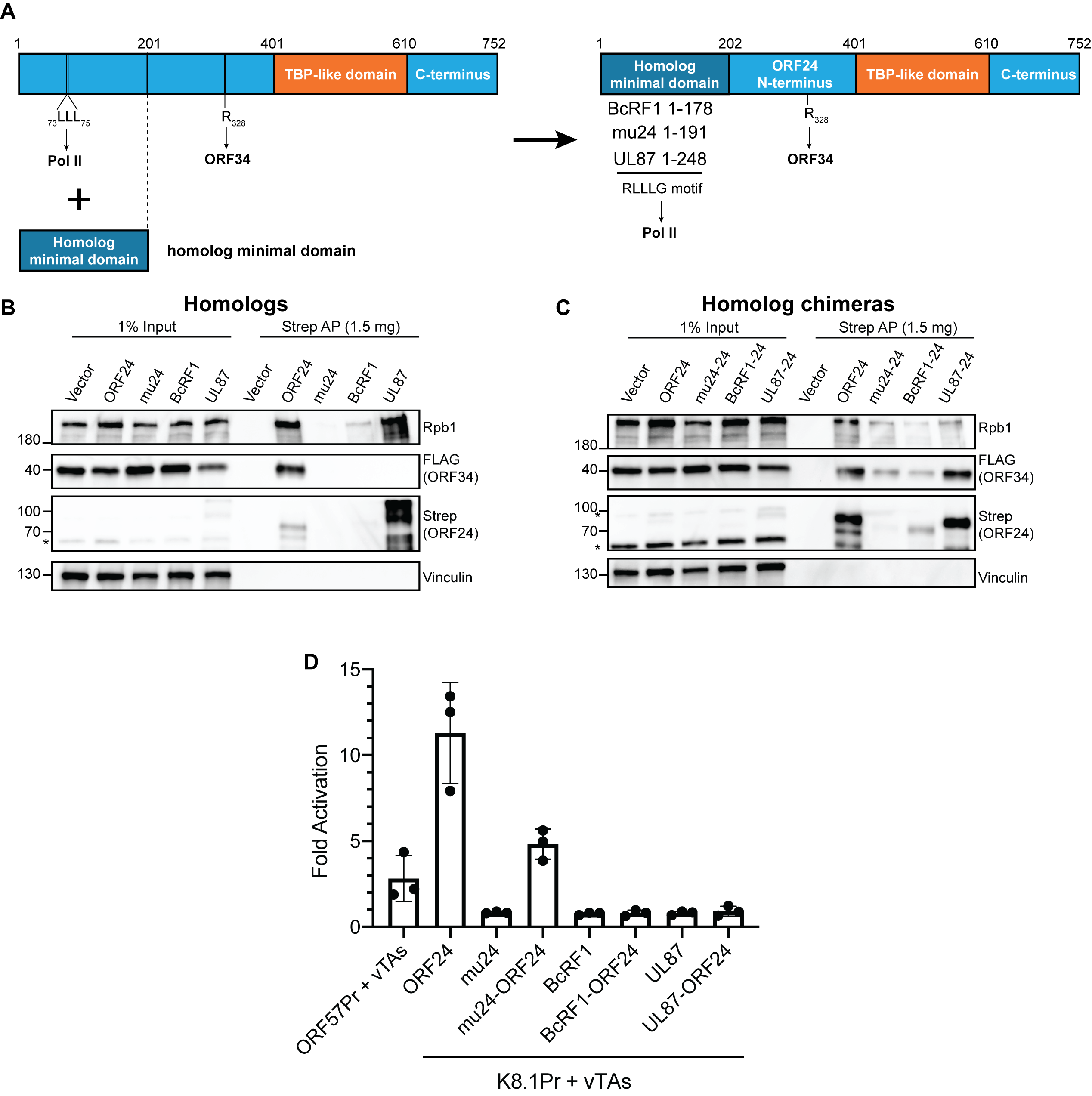

Supplement: S3 Fig — (A) Schematic of construct design for ORF24 chimeras. The ORF24-NTD (a.a. 1–201) was replaced with the experimentally identified minimal domain of mu24, BcRF1, and UL87. These chimeric constructs retain the N-terminal ORF24-ORF34 interaction region, the ORF24 vTBP domain, and ORF24 C-terminal tail. (B) Full-length Strep-tagged homologs of ORF24 were transiently transfected into HEK293T cells along with FLAG-tagged ORF34, then co-affinity purified with StrepTactinXT beads (AP) followed by western blotting. (*) indicates the presence of a non-specific band seen while using the anti-Strep antibody. (C) Full-length Strep-tagged chimeras of ORF24 were transiently transfected into HEK293T cells along with FLAG-tagged ORF34, then co-affinity purified with StrepTactinXT beads (AP) followed by western blotting. (*) indicates the presence of a non-specific band seen while using the anti-Strep antibody. (D) HEK293T cells were transiently transfected with a pGL4.16 firefly luciferase plasmid driven by either the ORF57 (early gene) or K8.1 (late gene) promoter. Plasmids encoding either ORF24, its homologs, or the chimeras, along with the five remaining KSHV vTAs (ORFs 18, 30, 31, 34, and 66) and a pRL-TK renilla luciferase plasmid (as a transfection control) were also transfected. After 24 h, cell lysates were harvested and luciferase activity was measured. Fold activation was calculated by normalizing to the firefly/renilla signal in the absence of vTAs. (TIF) [file ppat.1008843.s003.tif]

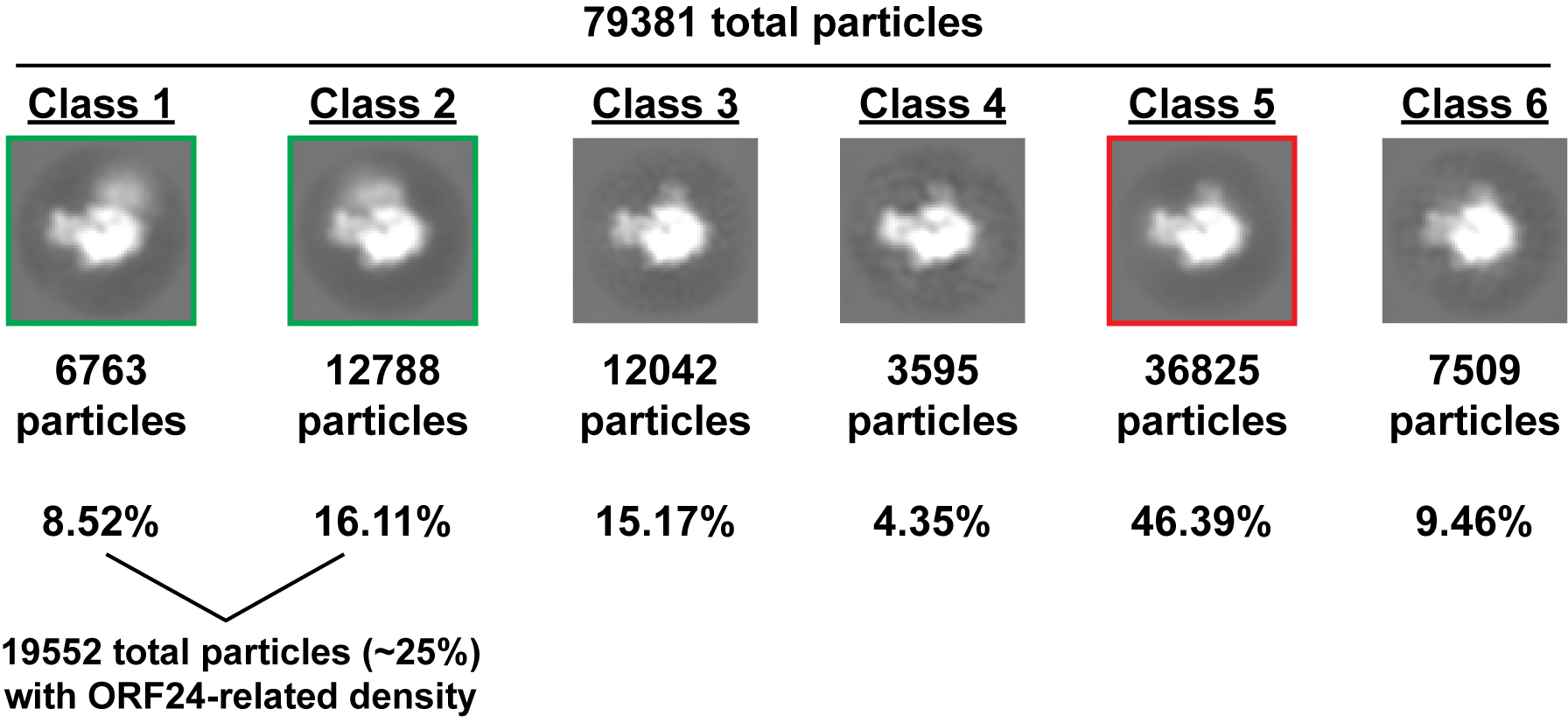

Supplement: S4 Fig — Two-dimensional projections of the three-dimensional classes resulting from sorting the single particle EM images of negatively stained minimal PICs (TBP/TFIIA/TFIIB/TFIIF/Pol II/DNA) assembled in the presence of GST-ORF24-NTD. The number of particles assigned to each class and the percent of total particles are indicated. Classes 1, 2, and 5 were used for the difference mapping shown in Fig 4. (TIF) [file ppat.1008843.s004.tif]
