# Supplementary material for: The gammaherpesviral TATA-box-binding protein directly interacts with the CTD of host RNA Pol II to direct late gene transcription
Source: PLoS Pathog. 2020 Sep 4;16(9):e1008843. doi: 10.1371/journal.ppat.1008843 (PMC7498053; doi:10.1371/journal.ppat.1008843)
Supplement: S1 Table — (DOCX) [file ppat.1008843.s005.docx]

**S1 Table. List of oligonucleotides used in this study**

| **#** | **Name** | **Sequence 5'-3'** | **Construct** |
| --- | --- | --- | --- |
| **1** | ORF24 1-133_pCDNA_F | GCTCGGATCCATGGCAGCGCTCGAGGG | pCDNA4.TO-ORF24 1-133-CFLAG |
| **2** | ORF24 1-133_pCDNA_R | TCGAGCGGCCGCCCACAATCATCGGTAAGTTCCCATGATC | pCDNA4.TO-ORF24 1-133-CFLAG |
| **3** | ORF24 1-201_pCDNA_F | GCTCGGATCCATGGCAGCGCTCGAGGG | pCDNA4.TO-ORF24 1-201-CFLAG |
| **4** | ORF24 1-201_pCDNA_R | TCGAGCGGCCGCCCCTCCAGGAGTGCAAAATAATTTTGATAGATTG | pCDNA4.TO-ORF24 1-201-CFLAG |
| **5** | ORF24 1-271_pCDNA_F | GCTCGGATCCATGGCAGCGCTCGAGGG | pCDNA4.TO-ORF24 1-271-CFLAG |
| **6** | ORF24 1-271_pCDNA_R | TCGAGCGGCCGCTTCTTGACGTCCTGGTGCTTACTCT | pCDNA4.TO-ORF24 1-271-CFLAG |
| **7** | ORF24 202-752_pCDNA_F | ATGAGCCTGAAGCATCTCTCG | pCDNA4.TO-ORF24 202-752-CFLAG |
| **8** | ORF24 202-752_pCDNA_R | GGATCCGAGCTCGGTACCAAG | pCDNA4.TO-ORF24 202-752-CFLAG |
| **9** | ORF24 1-201_pGEX_F | GCGTGGATCCATGGCAGCGCTCGAGG | pGEX4T1-ORF24-NTD |
| **10** | ORF24 1-201_pGEX_R | CGATGCGGCCGCTTACTCCAGGAGTGCAAAATAATTTTGATAGATTGTG | pGEX4T1-ORF24-NTD |
| **11** | ORF24 1-201_pMAL_F | ATTCGAGCTCAATGGCAGCGCTCGAGGG | pMAL-c2x-ORF24-NTD, WT and ΔLLL |
| **12** | ORF24 1-201_pMAL_R | TAGAGGATCCTTACTCCAGGAGTGCAAAATAATTTTGATAGATTGT | pMAL-c2x-ORF24-NTD, WT and ΔLLL |
| **13** | Rpb1_Linker aa 1460-1585_F | GCGTGGATCCCTGGGCCAGCTGGCTC | pGEX4T1-Rpb1-linker |
| **14** | Rpb1_Linker aa 1460-1585_R | CGATGCGGCCGCTGGTGAAGGGATGTAGGGGCT | pGEX4T1-Rpb1-linker |
| **15** | Rpb4 pGEX4T1_F | GGTTCCGCGTGGATCCATGGCGGCGGGTGG | pGEX4T1-hsRpb4 |
| **16** | Rpb4 pGEX4T1_R | GGAATTCCGGGGATCTTAATACTGAAAGCTGCGCT | pGEX4T1-hsRpb4 |
| **17** | Rpb7 pGEX4T1_F | CCCGGAATTCaggaggTAATTAAATatgttctaccatatctccctagagcac | pGEx4T1-hsRpb4/7 |
| **18** | Rpb7 pGEX4T1_R | CGATGCGGCCGCttagtgatggtggtgatggtggcttacaagccccaagtaatcgt | pGEX4T1-hsRpb4/7 |
| **19** | ORF24_2-201_p6H-SUMO3_F | cagcagacgggagggGCAGCGCTCGAGGGC | p6H-SUMO3-ORF24-NTD-Strep |
| **20** | ORF24_2-201_p6H-SUMO3_R | gacggagctcgaattTTATTTTTCAAACTGCGGATGGCTCCACTCCAGGAGTGCAAAATAATTTTGATAGATTG | p6H-SUMO3-ORF24-NTD-Strep |
| **21** | 2xCTD_repeat_pQLink_F | TTATTTTCAGGGATCCTATTCTCCGACCTCTCCATCATACAGCCCTACCTCCCCGTCCTAGGCGGCCGCCTAGGACCC | pQLink-GST-2xCTD |
| **22** | 2xCTD_repeat_pQLink_R | GGGTCCTAGGCGGCCGCCTAGGACGGGGAGGTAGGGCTGTATGATGGAGAGGTCGGAGAATAGGATCCCTGAAAATAA | pQLink-GST-2xCTD |
| **23** | 4xCTD_repeat_pQLink_F | TTATTTTCAGGGATCCTATAGCCCCACGAGCCCTTCTTACTCTCCGACCTCACCATCCTATTCGCCTACTAGCCCGAGTTACAGTCCCACATCTCCGTCCTAGGCGGCCGCCTAGGACCC | pQLink-GST-4xCTD |
| **24** | 4xCTD_repeat_pQLink_R | GGGTCCTAGGCGGCCGCCTAGGACGGAGATGTGGGACTGTAACTCGGGCTAGTAGGCGAATAGGATGGTGAGGTCGGAGAGTAAGAAGGGCTCGTGGGGCTATAGGATCCCTGAAAATAA | pQLink-GST-4xCTD |
| **24** | ORF24_F | TACCGAGCTCGGATCCATGGCAGCGCTCGAGGGC | pCDNA4.TO-ORF24 3L_A-CSTREP |
| **25** | ORF24_R | caccgcctccctcgaggacCAGCGGACG | pCDNA4.TO-ORF24 3L_A-CSTREP |
| **26** | BcRF1_F | TACCGAGCTCGGATCCATGACACAAGGTAAGAGGGAGATGG | pCDNA4.TO-BcRF1-CSTREP |
| **27** | BcRF1_R | CACCGCCTCCCTCGAGCACTTGAGCATCACGGCAGTGG | pCDNA4.TO-BcRF1-CSTREP |
| **28** | BcRF1 (3L_A)_F | GCCGCGGCAggcgttatccgaataaatgactgccaggag | pCDNA4.TO-BcRF1 3L_A-CSTREP |
| **29** | BcRF1 (3L_A)_R | acgacgcatagcccgacacgcgtaaaaggtg | pCDNA4.TO-BcRF1 3L_A-CSTREP |
| **30** | mu24_F | TACCGAGCTCGGATCCATGACAATATTCTTACCAGTATTCTGTGATTTGC | pCDNA4.TO-mu24-CSTREP |
| **31** | mu24_R | CTCCCTCGAGCGGCCGCCCGGAGTCTGGTTGGCAAGG | pCDNA4.TO-mu24-CSTREP |
| **32** | mu24 (3L_A)_F | GCCGCGGCAggcataagactgtacccaacctgcaatactg | pCDNA4.TO-mu24 3L_A-CSTREP |
| **33** | mu24 (3L_A)_R | tcttcttaaagctctacataaacagaatgt | pCDNA4.TO-mu24 3L_A-CSTREP |
| **34** | UL87_F | AAAAGAATTCATGGCCGGCGCTGC | pCDNA4.TO-UL87-CSTREP |
| **35** | UL87_R | AAAACTCGAGTCGTGATGCAAACCGCAC | pCDNA4.TO-UL87-CSTREP |
| **36** | UL87 (3L_A)_F | GCCGCGGCAGGACCCGTGGCCGTACCCTGTTTTTGCGAC | pCDNA4.TO-UL87 3L_A-CSTREP |
| **37** | UL87 (3L_A)_R | ACGACCCAGTACCAGCTTGACACGCTCGGA | pCDNA4.TO-UL87 3L_A-CSTREP |
| **38** | ORF24_1-191_pCDNA_F | TACCGAGCTCGGATCCATGGCAGCGCTCGAGGGC | pCDNA4.TO-ORF24 1-191-CSTREP |
| **39** | ORF24_1-191_pCDNA_R | CACCGCCTCCCTCGAGTGTGGGTTGTAGACTATGGGGC | pCDNA4.TO-ORF24 1-191-CSTREP |
| **40** | ORF24_1-201_pCDNA_F | TACCGAGCTCGGATCCATGGCAGCGCTCGAGGGC | pCDNA4.TO-ORF24 1-201-CSTREP |
| **41** | ORF24_1-201_pCDNA_R | CACCGCCTCCCTCGAGCTCCAGGAGTGCAAAATAATTTTG | pCDNA4.TO-ORF24 1-201-CSTREP |
| **42** | ORF24_1-226_pCDNA_F | TACCGAGCTCGGATCCATGGCAGCGCTCGAGGGC | pCDNA4.TO-ORF24 1-226-CSTREP |
| **43** | ORF24_1-226_pCDNA_R | CACCGCCTCCCTCGAGGTTAAACTTTAAAAAATGTAGC | pCDNA4.TO-ORF24 1-226-CSTREP |
| **44** | BcRF1_1-168_pCDNA_F | TACCGAGCTCGGATCCATGACACAAGGTAAGAGGGAGATGG | pCDNA4.TO-BcRF1 1-168-CSTREP |
| **45** | BcRF1_1-168_pCDNA_R | CACCGCCTCCCTCGAGGGTCCCTAGGAAGCGACGC | pCDNA4.TO-BcRF1 1-168-CSTREP |
| **46** | BcRF1_1-178_pCDNA_F | TACCGAGCTCGGATCCATGACACAAGGTAAGAGGGAGATGG | pCDNA4.TO-BcRF1 1-178-CSTREP |
| **47** | BcRF1_1-178_pCDNA_R | CACCGCCTCCCTCGAGAGCCAGCATCTTTGCAGAGTTTTGC | pCDNA4.TO-BcRF1 1-178-CSTREP |
| **48** | BcRF1_1-203_pCDNA_F | TACCGAGCTCGGATCCATGACACAAGGTAAGAGGGAGATGG | pCDNA4.TO-BcRF1 1-203-CSTREP |
| **49** | BcRF1_1-203_pCDNA_R | CACCGCCTCCCTCGAGGAAGTGGAACTTGAGTCTGGCC | pCDNA4.TO-BcRF1 1-203-CSTREP |
| **50** | mu24_1-181_pCDNA_F | TACCGAGCTCGGATCCATGACAATATTCTTACCAGTATTC | pCDNA4.TO-mu24 1-181-CSTREP |
| **51** | mu24_1-181_pCDNA_R | CACCGCCTCCCTCGAGAGCAGACCTGGCCCAGGG | pCDNA4.TO-mu24 1-181-CSTREP |
| **52** | mu24_1-191_pCDNA_F | TACCGAGCTCGGATCCATGACAATATTCTTACCAGTATTC | pCDNA4.TO-mu24 1-191-CSTREP |
| **53** | mu24_1-191_pCDNA_R | CACCGCCTCCCTCGAGCTCAGTAAGCTTCAAGTAATTTTTT | pCDNA4.TO-mu24 1-191-CSTREP |
| **54** | mu24_1-216_pCDNA_F | TACCGAGCTCGGATCCATGACAATATTCTTACCAGTATTC | pCDNA4.TO-mu24 1-216-CSTREP |
| **55** | mu24_1-216_pCDNA_R | CACCGCCTCCCTCGAGATTGAATTTGATATATTCCAAATGA | pCDNA4.TO-mu24 1-216-CSTREP |
| **56** | UL87_1-238_pCDNA_F | TACCGAGCTCGGATCCATGGCCGGCGCTGCGCCG | pCDNA4.TO-UL87 1-238-CSTREP |
| **57** | UL87_1-238_pCDNA_R | CACCGCCTCCCTCGAGGCGCGGCGCCGCGGCCTCG | pCDNA4.TO-UL87 1-238-CSTREP |
| **58** | UL87_1-248_pCDNA_F | TACCGAGCTCGGATCCATGGCCGGCGCTGCGCCG | pCDNA4.TO-UL87 1-248-CSTREP |
| **59** | UL87_1-248_pCDNA_R | CACCGCCTCCCTCGAGTCCAAAGAGCAGCTTGTAGTGAACC | pCDNA4.TO-UL87 1-248-CSTREP |
| **60** | UL87_1-273_pCDNA_F | TACCGAGCTCGGATCCATGGCCGGCGCTGCGCCG | pCDNA4.TO-UL87 1-273-CSTREP |
| **61** | UL87_1-273_pCDNA_R | CACCGCCTCCCTCGAGAAGCTTTTGCAGCTCCAGC | pCDNA4.TO-UL87 1-273-CSTREP |
| **62** | BcRF1-ORF24_1_F | TACCGAGCTCGGATCCATGACACAAGGTAAGAGGGAGATGG | pCDNA4.TO-BcRF1-ORF24-CSTREP |
| **63** | BcRF1-ORF24_1_R | TTCAGGCTagccagcatctttgc | pCDNA4.TO-BcRF1-ORF24-CSTREP |
| **64** | BcRF1-ORF24_2_F | gctggctAGCCTGAAGCATC | pCDNA4.TO-BcRF1-ORF24-CSTREP |
| **65** | BcRF1-ORF24_2_R | CACCGCCTCCCTCGAGCAGCGGACGGACGCAACG | pCDNA4.TO-BcRF1-ORF24-CSTREP |
| **66** | mu24-ORF24_1_F | TACCGAGCTCGGATCCATGACAATATTCTTACCAGTATTC | pCDNA4.TO-mu24-ORF24-CSTREP |
| **67** | mu24-ORF24_1_R | TTCAGGCTctcagtaagcttcaagtaattttt | pCDNA4.TO-mu24-ORF24-CSTREP |
| **68** | mu24-ORF24_2_F | tactgagAGCCTGAAGCATCTCT | pCDNA4.TO-mu24-ORF24-CSTREP |
| **69** | mu24-ORF24_2_R | CACCGCCTCCCTCGAGCAGCGGACGGACGCAACG | pCDNA4.TO-mu24-ORF24-CSTREP |
| **70** | UL87-ORF24_1_F | TACCGAGCTCGGATCCATGGCCGGCGCTGCGCCG | pCDNA4.TO-UL87-ORF24-CSTREP |
| **71** | UL87-ORF24_1_R | TTCAGGCTtccaaagagcagct | pCDNA4.TO-UL87-ORF24-CSTREP |
| **72** | UL87-ORF24_2_F | AGCCTGAAGCATCTCTCGTTTTCAAT | pCDNA4.TO-UL87-ORF24-CSTREP |
| **73** | UL87-ORF24_2_R | CACCGCCTCCCTCGAGCAGCGGACGGACGCAACG | pCDNA4.TO-UL87-ORF24-CSTREP |
