# Supplementary material for: The gammaherpesviral TATA-box-binding protein directly interacts with the CTD of host RNA Pol II to direct late gene transcription
Source: PLoS Pathog. 2020 Sep 4;16(9):e1008843. doi: 10.1371/journal.ppat.1008843 (PMC7498053; doi:10.1371/journal.ppat.1008843)
Supplement: S2 Table — (DOCX) [file ppat.1008843.s006.docx]

**S2 Table. Nucleotide sequence of synthetic gene blocks used in this study.**

| **Geneblock Name** | **Sequence 5'-3'** | **Construct** |
| --- | --- | --- |
| 10xCTD_repeat_pQLink_insert | TTATTTTCAGGGATCCTATAGCCCGACGTCGCCGAGTTATTCACCTACGTCCCCATCATACTCCCCCACGAGCCCTAGTTATTCGCCAACTTCCCCGAGCTATTCCCCAACATCACCCAGCTATAGTCCCACTTCACCCTCCTATTCACCTACGAGTCCATCTTATTCTCCAACCAGTCCTTCGTACTCACCCACGTCCCCATCGTATTCTCCTACTTCCCCTAGCTAGGCGGCCGCCTAGGACCC | pQLink-GST-10xCTD |
