# Supplementary material for: The gammaherpesviral TATA-box-binding protein directly interacts with the CTD of host RNA Pol II to direct late gene transcription
Source: PLoS Pathog. 2020 Sep 4;16(9):e1008843. doi: 10.1371/journal.ppat.1008843 (PMC7498053; doi:10.1371/journal.ppat.1008843)
Supplement: Attachment — Submitted filename: 20200723_PlosPathogens_response-to-reviewers_BG_ALD.docx [file ppat.1008843.s007.docx]

**Response to Reviewers**

**Part I – Summary**

*Reviewer #1: The authors have examined the interaction between vTBP proteins (ORF24, mu24, UL87, BcRFI) expressed in beta and gamma herpesviruses that interact with the late gene TATT promoter element and RNA polymerase II. They conclude that a highly conserved stretch of 5 amino acids (RLLLG) in the N-terminal region of the vTBPs is responsible for binding directly to the CTD of the large subunit of RNA polymerase II when it is hypophosphorylated. The results strongly support this conclusion, but it is this reviewer’s opinion that few important new findings have been made. An important Molecular Cell paper (2015) on ORF24 from the same lab demonstrated an RLLLG-dependent interaction between ORF24 and Pol II. In that paper they demonstrated that the interactions was disrupted when the CTD was phosphorylated. They report here a small, incremental finding that the RLLLG-dependent interaction is with the CTD and that that interaction is at least in part direct.*

We disagree that our finding that the ORF24 vTBP directly contacts the CTD of Pol II is an incremental discovery. While we previously demonstrated that ORF24 coimmunoprecipitates with the hypophosphorylated 12-subunit Pol II in cells, we had no evidence that this was a direct interaction or, if so, which of the Pol II subunits might mediate contact. Indeed, an indirect interaction might have been the more likely scenario, given that the phosphorylation state of the CTD affects interactions with numerous complex RNA processing machines and cellular TBP does not directly contact Pol II. Only two other viral proteins are known to directly contact Rpb1 (HSV-1 ICP27 and the bat influenza A polymerase FluPol), and FluPol is the sole viral protein whose Pol II CTD interaction has been structurally characterized. Our EM visualization of ORF24 NT bound to a purified, *in vitro* assembled PIC and biochemical validation of the ORF24-CTD interaction provides significant insight into the noncanonical mechanism of ORF24-directed Pol II loading.

Specific Comments: *1. There are claims that vTBP is the only know transcription factor that interacts with DNA and Pol II. They say, “We conclude that vTBP is a fundamentally unique protein when compared to other eukaryotic Pol II-interacting proteins, as it both directly interacts with the Pol II CTD and binds promotor DNA to coordinate late gene expression.” It is true that the carboxyl terminal half of the 339 aa human TBP used for structural studies does not appear to interact directly with Pol II. However, TFIIB interacts with the DNA surrounding the TBP binding site and interacts with Pol II. The vTBP is about 3 times larger than TBP with other critical domains not contained within TBP. The goal of bridging the promoter DNA to Pol II is accomplished in the host and virus through the coordinated interactions of DNA binding proteins, Pol II binding proteins and Pol II. There is no reason to expect vPICs would operate using the same connectivity as found the host especially given the large differences in the proteins used.*

ORF24 contains a TBP-like domain that directly binds that TATT promoter element; here we show that vTBP also directly interacts with Pol II. There are no known eukaryotic proteins that have both of these functions. TFIIB binds the ‘BRE’ element, not the TATA motif. Indeed, we do not expect the vPIC to have the same connectivity as human PICs- we expect TBP to be replaced by vTBP, and potentially other GTFs to be replaced by vTAs. Our conclusion that “vTBP is a fundamentally unique protein when compared to other eukaryotic Pol II-interacting proteins” is supported by this manuscript and previous work. To clarify, we have added “binds [the core late gene] promoter to coordinate late gene expression” (line 114).

*2. In 2B what are the proteins in the ~100 kDa range in the inputs? Why is the full length protein not expressed well?*

In all of our westerns, we observe appearance of two nonspecific bands that show up in the ~70 kDa and ~100 kDa range which we have labeled on all of the relevant figures (Figs 1, 2, 3, new S2, S3); these bands are visible because of the poor expression of vTBP. We have now replaced the original Figure 1 with a new version where we have shown each WT and mutant vTBP homolog separately to better optimize the exposure for the westerns. Full-length vTBP and its homologs (particularly mu24 and BcRF1) are poorly expressed for unknown reasons, though the fact that ORF24 is poorly expressed even in the context of the virus (Nandakumar *et al.* 2019; Didychuk *et al.* 2019), suggests that this is an inherent property of the protein and may be important for its regulation *in vivo*.

*Why is Pol II pulled down much more efficiently by the full length protein (more than 10 times better)? Are other domains critically important?*

Based on Fig. 2 and other data not shown, no other domain of ORF24 (i.e. residues 202-752) directly contributes to the Rpb1 interaction. Based on our experience with the BcRF1 fragments, which by themselves do not interact with Rpb1 despite better expression than full-length protein, but do interact when fused to the rest of ORF24 (Fig. S3), it is likely that truncation of the protein changes some property of the protein or destabilizes it, preventing interaction with Pol II. This trend also fits with the observation that the fragments of all homologs, which express better than their respective full-length proteins, pull down comparatively less Rpb1.

*Pol II CTD should not be the label for the westerns it should be RPB1 or something to indicate it is the large subunit of Pol II.*

We labeled the westerns with “Pol II CTD” to reflect that we use a monoclonal antibody directed against the Pol II CTD, but have changed this to Rpb1 in all relevant figures to prevent confusion.

*Why are there no phosphorylated forms of the large subunit seen in the input?*

The anti-Pol II 8WG16 clone detects multiple phosphorylation states of the CTD, but these states are not distinguished based on size and are typically distinguished by the use of a monoclonal antibody that detects a specific phosphorylation state.

*3. In 3B NTDs of ORF24 and UL87 do pull our Pol II, but MHV-68 (mu24) and BcRF1 do not. (this is not what was said).*

The mu24-Rpb1 interaction for the mu24 fragments is very weak, but can be distinguished from background at optimal exposure (Fig. 3B). We have included an additional supplementary figure with a longer exposure of Rpb1 to highlight this (new S2B Fig). In the discussion for this figure we state that “In contrast, all truncations of BcRF1 failed to interact with Pol II (Fig 3C).” (lines 179-180). However, when these BcRF1 fragments are fused to ORF24 202-752, they are capable of interacting with Rpb1: “Interestingly, although the minimal domain of BcRF1 alone failed to interact with Pol II by co-IP (Fig 3D), it is capable of interaction with Pol II when fused to ORF24 (S2C Fig)” (lines 195-197).

*4. It is not clear to me what important was learned from the chimera studies.*

Many groups working on late gene transcription in the β- and γ-herpesviruses have shown that the vTA complex is exquisitely sensitive to perturbation, and that while the vTA complex may be functionally conserved, no individual vTA can be exchanged for its homolog (likely due to extensive protein-protein interactions in the complex). It is significant to the field when any domain swapping is even partially functional, as it suggests that the domain is at least partially functionally isolated from the remainder of the vTA complex. These types of experiments help tease apart the functions of the vTAs.

*5. I could not figure out why negative stain EM was used to examine the interaction of the domain of vTBP with the partial host PIC complex containing TBP. The previous published studies already strongly pointed to the CTD mediating the interaction between vTBP and Pol II. Clearly, this is not a physiologically relevant complex.*

Actually, no prior studies addressed either the region or subunit of Pol II involved in the vTBP interaction nor whether the interaction with the Pol II complex was direct or indirect; they simply established that the vTBP preferentially binds the Pol II complex in its unphosphorylated state. The Rpb1 antibody used in those IPs served as a proxy for the Pol II complex as a whole, rather than an indicator of which subunit mediated the interaction, as all 12 subunits together co-IP with ORF24 (Davis *et al.* 2015). Although the PIC we visualized by negative stain EM could not examine the interaction of the vTBP with DNA (due to ongoing challenges with expressing and purifying full-length ORF24), it was nonetheless quite valuable in that it revealed that the interaction with Pol II is direct and allowed us to use subsequent biochemistry to identify the interaction site.

*In conclusion, the study was performed to high standards, but failed to deliver a satisfactory level of new information.*

We thank the reviewer for their positive assessment of the data quality, but respectfully disagree with the conclusion that we have failed to glean new mechanistic insights. We have: (1) isolated the region of the protein that is necessary for the interaction between vTBP and Pol II, (2) extended these results to other medically important herpesviruses, and (3) refined the region that vTBP interacts with in the 550 kDa Pol II complex to 4xCTD repeats. Establishing that a protein-protein interaction occurs in lysate was a valuable first step, and here we have significantly advanced our mechanistic understanding of this interaction using *in vitro* reconstitution.

*Reviewer #2: Beta and gamma herpesviruses require at least seven virally encoded proteins to orchestrate transcription of late viral genes. These late gene regulators (or viral transcription activators vTAs) assemble into a viral transcription pre-initiation complex (vPIC) on late gene promoters. One essential component of this complex is the viral TATA-box binding protein (vTBP) that recognizes a TATT element present in late promoters. A previous study from the same group demonstrated the capacity of vTBP homologs to interact with RPB1, the catalytic subunit of RNA polymerase II. The interaction was mediated by three leucine residues (RLLLG motif) in KSHV-TBP (orf24). This presents an interesting model in which vTBPs are directly involved in recruitment of RNA polymerase to transcribe late genes. Such model would be different from transcription of cellular genes in which the host TBP does not directly interact with RNAPII. However, to establish this model two essential questions had to be addressed: does vTBP directly interact with subunits of RNAPII? Which subunit of RNAPII mediates the interaction? In the current manuscript the authors addressed these questions and made the following novel observations:
1) the RLLLG motif, which is conserved among beta and gamma-herpesviruses, mediates the interaction with RPB1.
2) The N-terminal domain of ORF24 (KSHV TBP) homologs is necessary and sufficient for interaction with RPB1.
3) Using single particle negative stain EM, the authors assembled a minimal PIC containing GST-ORF24-NTD and were able to obtain structural information on the NTD of ORF24 bound to RNAPII. This information is crucial as it shows a direct interaction between ORF24 and RNAPII. Two potential interaction interfaces were determined using EM; these are RPB4/7 and RPB1-CTD.
4) Using GST-pulldown assays the authors demonstrated that interaction of ORF24-NTD with CTD requires at least four heptad repeats. No interaction was observed between ORF24-NTD and RPB4/7. Overall, the manuscript is well written, the topic is important, the experiments are well executed and the results are clear and support the conclusions.*

We thank the reviewer for their concise and accurate summary of our results and previous work.

*Reviewer #3: In this manuscript, Castañeda et al. describes the studies on the interactions between ORF24 of KSHV, a herpesviral TBP and cellular RNA polymerase II (PolII). First, they extended the observation that they previous made on KSHV ORF24 to other herpesival homologues encoded by MHV68, ENV, and HCMV. A conserved leucine-rich motif required for KSHV ORF24 to interact with RNA PolII is also critical for other homologues. Second, they determined the N-terminal domain that contains the leucine-rich motif is sufficient to bind PolII. Third, the in vitro assembled ORF24-PolII minimal PIC complex was examined by EM and the potential regions of PolII interactions were identified. One region, the CTD repeats of Rbp1, was confirmed by GST-pulldown and concluded by the authors to be the primary contact point. Cellular TBP does not bind PolII directly; instead, the recruitment of PolII is mediated by TFIIB or other GTFs. Therefore, the major contribution from this manuscript is the demonstration of a direct interaction between a herpesviral TBP and PolII. The manuscript is well written and easy to read.*

We thank the reviewer for their assessment of our manuscript.

**Part II – Major Issues: Key Experiments Required for Acceptance**

Reviewer #1: (No Response)

Reviewer #2: None

Reviewer #3:

*1. There should be a clear description of the rationale using cellular TBP in the in vitro assembly described in Fig. 4 for EM studies, especially because TBP is not part of vPIC as this group previously determined. An explanation of why a TBP-like containing ORF24 and a viral TATA promoter is not used. Has this been attempted but without success? Since only the N-terminal domain of ORF24 without TBP-like domain was used and instead cellular TBP was used in the assembly, a discussion on limitations of the EM result needs to be included. For example, is it expected that the TBP-like domain of ORF24 would occupy the same position as cellular TBP? If not, how this would affect the EM structural results?*

We agree that the rationale for this experiment should be further clarified and have included additional text (232-233 and 239-246). Indeed, while we are highly enthusiastic about including full length ORF24 in this assay, at this point (despite several years of effort) it has remained intractable to purification.

*2. From Fig.1 to Fig.3, there were several mutant constructs that did not express well or efficiently immunoprecipitated. For example, the 3L mutant of mu24 (Fig. 1), the two mutants (Fig. 2), 202-752 and 1-133, as well as the 1-191 mutant (Fig. 3A), the 1-181 mutant (Fig. 3B) and the 1-203 mutant (Fig. 3C). The conclusion that these mutants do not interact with PolII cannot be drawn unless comparable immunoprecipitation or expression of the mutants is achieved.*

We have replaced Fig 1 with new westerns where we look at each homolog (WT vs. RAAAG) separately, which has allowed us to optimize exposure for each homolog. We have also included an additional supplemental figure (S2 Fig) where we modify the amount of DNA transfected for the ORF24 fragments such that ORF24 1-191 is the most abundant of the three fragments. Even in this case, it is clear that interaction with Pol II is eliminated with further truncation of the protein. We do not seek to make quantitative statements about the relative contribution of the remainder of the protein, especially given the caveats that occur when making truncations to the protein (as in the case of BcRF1). We used these experiments to motivate our *in vitro* experiments, and to highlight that the N-terminal domain is important in all homologs of ORF24.

**Part III – Minor Issues: Editorial and Data Presentation Modifications**
*Please use this section for editorial suggestions as well as relatively minor modifications of existing data that would enhance clarity.*

Reviewer #1: (No Response)

Reviewer #2:
*Fig 3: In line 172 the authors concluded that all truncations of BcRF1 failed to interact with RNAPII. However, BcRF1(1-203) is expressed at a very low level relative to other BcRF1 mutations. This might explain why the NTD of BcRF1 is insufficient to precipitate RPB1.*

Based on homology to ORF24, we would expect BcRF1(1-178), which is well-expressed, to interact with Pol II; however, we have not observed this interaction in any of our replicates. Interestingly, when BcRF1 1-178 is fused to ORF24 202-752 (Fig. S2C), it is able to interact with Rpb1, suggesting that the crucial domain is intact, but that truncation of the protein changes a property that prevents it from interacting with Rpb1.

*S2 Fig: Testing the ability of the NTD chimeras to functionally complement ORF24 was performed using a reporter assay. Perhaps the authors should consider using KSHV infected cells in which expression of the endogenous ORF24 was disrupted.*

While we agree that functionally complementing KSHV lacking ORF24 with the NTD chimeras would further support our data from the reporter assay, we felt that we would not gain further significant mechanistic insight from such experiments. The late gene reporter assay is well-established, and we have yet to identify mutations in the vTAs that do not have the same effect in the context of the virus as they do in the context of the reporter assay.

*Fig 4. Would the authors provide explanation on why there are two 3D classes (class 1 and 2) of high density on opposing faces of PolII observed with single particle negative stain EM?*

The CTD is highly flexible, and the two classes likely come from ORF24-NTD binding in multiple orientations relative to the rest of the PIC. We have included an additional sentence (line 256-258) to clarify this.

*Fig 4F: The authors used class 5 to subtract the density corresponding to the minimal PIC and to identify the region occupied by GST-ORF24-NTD. It is not clear how the authors concluded that class 5 represents minimal PIC that lacks ORF-24-NTD? Is it feasible to compare the density obtained from class 5 molecules with the density of corresponding molecules obtained in He et al (ref 30), assuming availability?*

Class 5 is nearly identical to the negative stain reconstruction of the TBP-TFIIA-DNA-TFIIB-Pol II-TFIIF complex as shown in Fig 1C of He *et al.* (2013). There are high resolution cryo-EM structures of this same complex, and all of the densities in the map for Class 5 in this paper can be accounted for by subunits of the minimal PIC complex (Fig 4E). The cryo-EM maps for the minimal PIC from He *et al.* include the DNA (which is not visible in the negative stain maps), and so we felt that a direct comparison of the maps would confuse non-experts. We have restructured a sentence in the discussion for Fig. 4 (line 250-254) to clarify that Class 5 in this manuscript corresponds to the known structure of the minimal PIC in He *et al*.

*Lines 273/274: To conclude that ORF24 does not interact with RPB4 or RPB7, the authors should consider examining the capacity of full length ORF24 to interact with these two subunits. So far the data suggests that the NTD of ORF24 does not interact with RPB4/7.*

Unfortunately, we are currently unable to generate full-length ORF24 in amounts sufficient to perform these *in vitro* experiments. We agree that our data show that the NTD of ORF24 does not interact with RPB4/7, but that it is possible that full-length ORF24 may interact with RPB4/7. We have updated the text to reflect this conclusion (line 273).

Reviewer #3: (No Response)
